# Supplementary material for: The causal relationship between the human gut microbiota and pyogenic arthritis: a Mendelian randomization study
Source: Front Cell Infect Microbiol. 2024 Nov 26;14:1452480. doi: 10.3389/fcimb.2024.1452480 (PMC11629706; doi:10.3389/fcimb.2024.1452480)
Supplement: Supplementary file 1 [file DataSheet1.zip › mendelian test/ebi-a-GCST90027498.csv_forest.pdf]

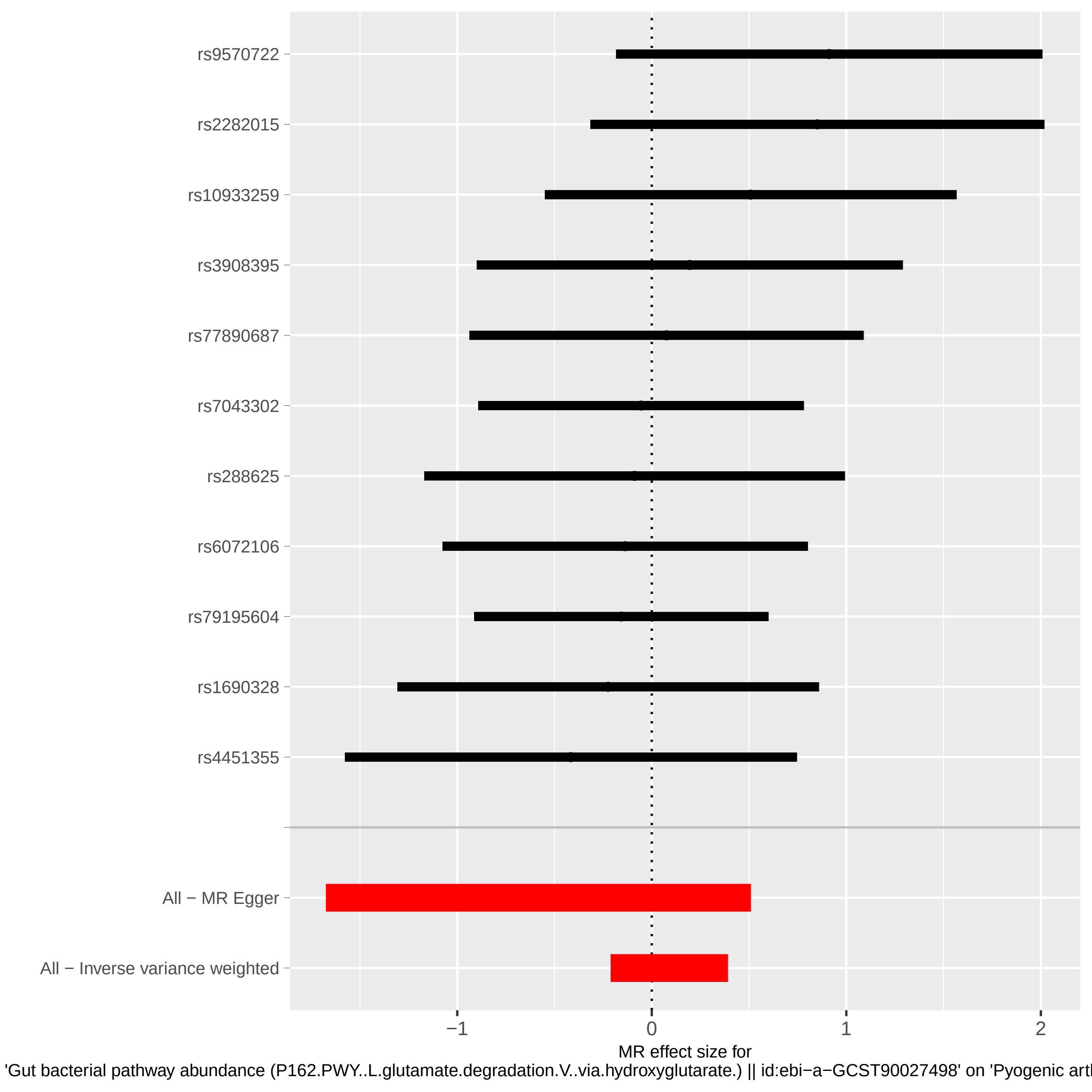

rs9570722
rs2282015
rs10933259
rs3908395
rs77890687
rs7043302
rs288625
rs6072106
rs79195604
rs1690328
rs4451355
All − MR Egger
All − Inverse variance weighted
−1
0
1
2
MR effect size for
'Gut bacterial pathway abundance (P162.PWY..L.glutamate.degradation.V..via.hydroxyglutarate.) || id:ebi−a−GCST90027498' on 'Pyogenic art
